# Outcome of stress on G protein-coupled receptors and hypoxia inducible factor-1α

**DOI:** 10.25122/jml-2023-0363

**Published:** 2024-02

**Authors:** Aldo Arturo Reséndiz-Albor, Ivonne Maciel Arciniega-Martínez, Arturo Contis Montes de Oca, Fabiola Guzmán-Mejía, Maria Elisa Drago-Serrano, Tania Estrada-Jiménez, Edgar Abarca-Rojano

**Affiliations:** 1 Laboratorio de Inmunidad de Mucosas, Escuela Superior de Medicina, Instituto Politécnico Nacional, Plan de San Luis y Díaz Mirón, Ciudad de México, México; 2 Sección de Estudios de Posgrado e Investigación, Escuela Superior de Medicina Instituto Politecnico Nacional Plan de San Luis y Salvador Diaz Miron, Ciudad de México, México; 3 Departamento de Sistemas Biológicos, Universidad Autónoma Metropolitana, Unidad Xochimilco, Ciudad de México, México; 4 Facultad de Medicina, Decanato de Ciencias Médicas, Universidad Popular Autónoma de Estado de Puebla, Ciudad de México, México

**Keywords:** HIF-1α, hypoxia, restraint stress, colon, GPR41/GPR43

## Abstract

Stress drives neuroendocrine signals with detrimental effects to the intestinal homeostasis. The aim of this study was to evaluate the effect of stress on intestinal hypoxia response elements, including G protein-coupled receptor 41 (GPR41), GPR43, and hypoxia inducible factor (HIF)-1α. Groups of five BALB/c mice were subjected to acute (2 h per day) and chronic (2 h per day for 4 days) stress induced by restraint, and the results were compared to those of an unstressed control group. Whole mucosal samples from the colon were collected to evaluate the expression of GPR41, GPR43 and HIF-1α using Western blot chemiluminescent analysis. Compared to the control group, in the chronic stress group the expression of GPR43 (*P* = 0.0092) and HIF-1α (*P* < 0.0001) were significantly lower and the expression of GPR41 was similar (*P* = 0.9184); acute stress significantly increased HIF-1α expression (*P* = 0.0030) and increased GPR41 expression (*P* = 0.0937), without affecting GPR43 (*P* = 0.9184). These findings offer insights into the modulation of hypoxia response elements under stress conditions and their pharmacological implications for developing drugs that mitigate the effects of stress on intestinal homeostasis.

## INTRODUCTION

Stress modulates intestinal inflammation through hormones released by the adrenal glands, such as catecholamines and glucocorticoids, regarded as endpoint markers of the activation of the hypothalamic–pituitary–adrenal axis [1]. The mechanisms through which stress affects the intestinal homeostasis may involve hypoxia inducible factor (HIF)-1α and G protein-coupled receptor 41 (GPR41) and GPR43 [2–5]. HIF-1αβ is a heterodimeric protein complex consisting of subunits HIF-1α and HIF-1β. In the presence of oxygen, HIF-1α is hydroxylated by prolyl hydroxylase (PHD) for ubiquitination and directed to the proteasome for degradation, whereas under conditions of oxygen depletion, PHD is inhibited, therefore HIF-1α hydroxylation and ubiquitination do not take place. HIF-1α is a sensor of hypoxia, whereas the HIF-1αβ complex acts as regulator of a wide array of metabolic processes under hypoxia [6]. GPR41 (also known as FFAR3) and GPR43 (known also as FFAR2) are a group of seven transmembrane protein receptors that regulate a wide array of events through signal pathways triggered by their interaction with ligands such as microbiota-derived short chain fatty acids (SCFAs) [7]. GPR41 and GPR43 ligands, such as butyrate, promote the stabilization of HIF-1α under hypoxic conditions and the regulation of homeostasis, as well as inflammation in the distal gut [8–10]. Thus, through the butyrate–HIF-1α pathway, butyrate provides protective signals in the intestinal milieu.

In experimental animal settings, a decrease in fecal levels of butyric and propionic acids, along with reduced colonic protein expression of GPR41, was observed in mice subjected to chronic stress induced by restraint and social disruption [2]. Additionally, increased levels of HIF-1α protein were detected in the oral cavity of rats with periodontitis exposed to chronic psychological stress [3], and in the gastric mucosa of rats subjected to acute water-immersion restraint stress [4]. Conversely, decreased HIF-1α mRNA levels were noted in the duodenum of mice under acute restraint stress [11]. To gain insights into the mechanisms through which stress modulates intestinal inflammation, this study aimed to assess the effect of acute and chronic stress on the expression of GRP41, GPR43 and HIF-1α.

## MATERIAL AND METHODS

### Experimental model

The study was conducted on two groups of five BALB/c mice (10–12 weeks old) subjected to acute (2 h) and chronic (2 h per day for 4 days) restraint stress. The results were compared to those of an unstressed control group, as described previously in detail [12].

### Western blot assay

Upon completing the stress protocols, whole mucosal samples from the colon were collected to evaluate the expression of GPR41, GPR43 and HIF-1α, and analyzed by Western blot assay, as following the method described previously in detail [12]. The target proteins were detected using antibodies anti-GRP41 (GeneTex), anti-GRP43 (GeneTex), and HIF-1α (GeneTex), and revealed with HRP-conjugated secondary antibodies (Invitrogen, Thermo Fisher Scientific). The experiments were repeated three times.

### Statistical analysis

Statistical analysis was performed using GraphPad Prism v.9 (GraphPad Software). Multiple comparisons among groups were analyzed using one way analysis of variance (ANOVA), and the means of each group were compared using post-hoc Tukey’s multiple comparison test. Results from one representative assay are expressed as mean ± s.d. *P* values of <0.05 were considered statistically significant.

## RESULTS

**Figure 1:**
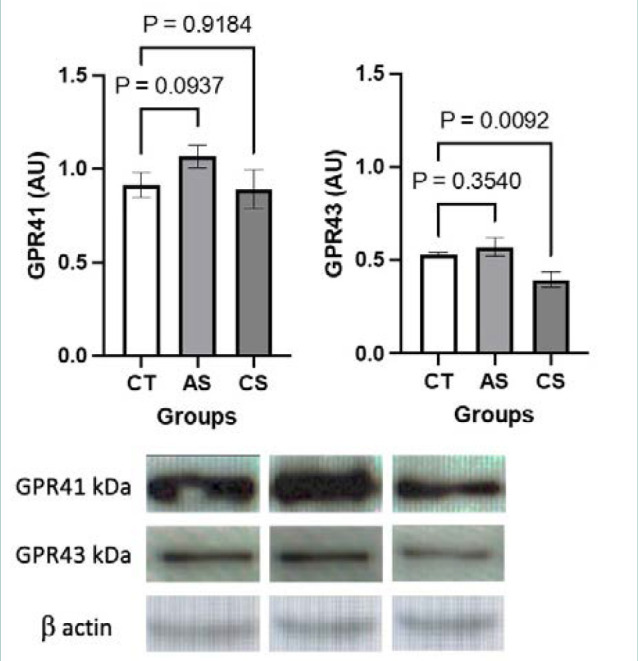
Representative Western blots of GPR41 and GPR43 in colonic mucosal samples of mice in the control (left), acute stress (middle) and chronic stress (right) groups. CT, control; AS, acute stress; CS, chronic stress. The results of densitometry analysis are expressed in arbitrary units (AU, y-axis) for each group (x-axis). The expression of the housekeeping protein β-actin is expressed as mean ± s.d., in AU (n = 5 mice per group).

**Figure 2:**
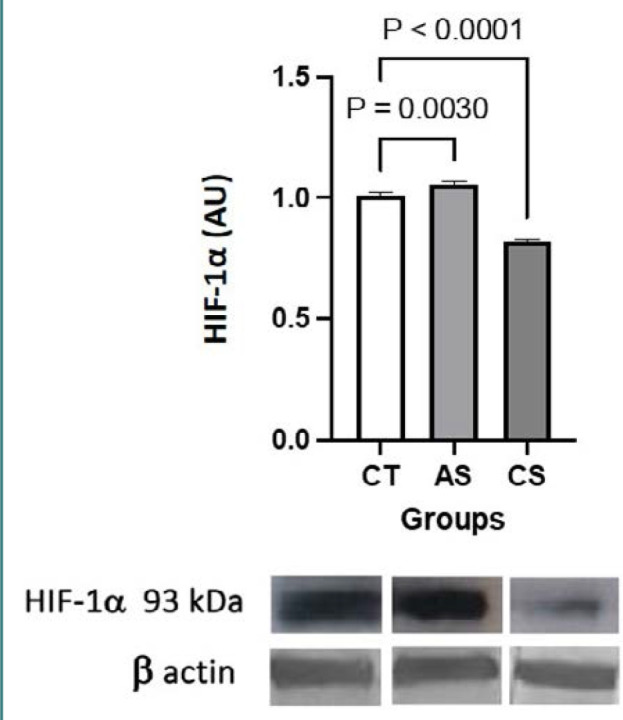
Representative Western blots of HIF-αβ in colonic whole mucosal samples of control (left), acute stress (middle) and chronic stress (right) groups. The results of densitometry analysis are expressed in arbitrary units (AU, y-axis) for each group (x-axis). The expression of the housekeeping protein β-actin is expressed as mean ± s.d., in AU (n = 5 mice per group).

Compared to the control group, GPR41 expression was higher in the acute stress group (*P* = 0.0937) and similar in the chronic stress group (*P* = 0.9184), whereas GRP43 expression was significantly lower in the chronic stress group (*P* = 0.0092) and apparently increased in the acute stress group (*P* = 0.3540) (Figure 1). Compared to the control group, HIF-1α expression was significantly higher in the acute stress group (*P* = 0.0030) and significantly lower in the chronic stress group (*P* < 0.0001) (Figure 2).

## DISCUSSION

Previous studies found that chronic stress decreased the expression of GPR41, without affecting GPR43 [2]. In this study, chronic stress downmodulated the expression of GPR43 and HIF-1α, without affecting GPR41. This may be attributed to different experiment settings; however, the findings suggest that stress has a negative effect on the abundance of microbiota involved in the release of SCFAs, such as *Lactobacilli* [2]. SCFAs act as endogenous HIF-1α PHD inhibitors, blocking the degradation of HIF-1α. In other words, SCFAs enhance the expression of HIF-1α via GPR41 and GPR43 signaling [8,9,13]. The downmodulation effect of chronic stress on GPR43 and HIF-1α protein expression may be linked with stress-induced corticosterone response, as reported previously [12]. As documented in in vitro HepG2 culture assays, dexamethasone has a detrimental effect on HIF-1α activity in a GPR-dependent manner. Under hypoxic conditions, dexamethasone promotes the sequestration of HIF-1α by cytosolic proteins, particularly heat shock proteins, thereby impeding the nuclear translocation of HIF-1α [5]. It is believed that HIF-1α acts as a regulator of stress-induced adrenal gland responses and therefore may be a potential target for therapeutic interventions [14].

In contrast to chronic stress, acute stress increased GPR41 and HIF-1α expression (the latter significantly) without affecting GPR43. These results may reflect a complex interplay between GPR, HIF-1α, and stress-induced catecholamine release. Catecholamines act as GPR ligands, resulting in HIF-1α stabilization [15], and promote bacterial growth by forming complexes with iron-binding proteins loaded with Fe3+ reduced to Fe2+ for bacterial uptake via receptors [16]. It is known that free iron chelates act as PHD inhibitors, promoting HIF-1α stabilization [17]. Accordingly, our findings suggest that the acute stress response involving catecholamines may promote bacterial growth, leading to the release of SCFAs, which interact with GPR41 and GPR43 involved in HIF-1α expression.

We are aware that the findings of this study raise more questions than answers, and many of these questions may be addressed in a future study. However, we are confident that these data offer insights into the modulation of hypoxia response elements under stress and their pharmacological implications for developing drugs that mitigate the effects of stress on intestinal homeostasis. Our next objectives involve assessing the bacterial count of microbiota members *Lactobacillus* and *Bifidobacterium* involved in SCFA metabolism, evaluating intracellular cytokines labeled pro- and anti-inflammatory in lymphoid cells, and examining catecholamine receptors in colonic samples of mice subjected to stress and fed a diet supplemented with butyric acid.

## Conclusion

This study found that chronic stress significantly decreased the expression of GPR43 and HIF-1α without affecting the expression of GPR41, whereas acute stress significantly increased the expression of HIF-1α and increased the expression GPR41, without affecting the expression of GPR43.

## Data Availability

Further data is available from the corresponding author on reasonable request.
